# Changing trends in reproductive/lifestyle factors in UK women: descriptive study within the UK Collaborative Trial of Ovarian Cancer Screening (UKCTOCS)

**DOI:** 10.1136/bmjopen-2016-011822

**Published:** 2017-03-06

**Authors:** Aleksandra Gentry-Maharaj, Clara Glazer, Matthew Burnell, Andy Ryan, Hannah Berry, Jatinderpal Kalsi, Robert Woolas, Steve J Skates, Stuart Campbell, Mahesh Parmar, Ian Jacobs, Usha Menon

**Affiliations:** 1 Department of Women's Cancer, Institute for Women's Health, UCL, London UK; 2 Department of Occupational and Environmental Medicine, Frederiksberg-Bispebjerg University Hospital, Copenhagen, Denmark; 3 Department of Gynaecological Oncology, Queen Alexandra Hospital, Portsmouth, UK; 4 Massachusetts General Hospital Biostatistics, Massachusetts General Hospital and Harvard Medical School, Boston, Massachusetts, USA; 5 Create Health Clinic, London, UK; 6 Medical Research Council Clinical Trials Unit at University College London, London, UK; 7 Centre for Women's Health, Institute of Human Development, University of Manchester, Manchester, UK; 8 University of New South Wales, Sydney, New South Wales, Australia

**Keywords:** trends, birth cohort, UKCTOCS, UK, postmenopausal women, reproductive factors

## Abstract

**Objective:**

There has been considerable interest in the impact of reproductive factors on health but there are little data on how these have varied over time. We explore trends in reproductive/lifestyle factors of postmenopausal British women by analysing self-reported data from participants of the UK Collaborative Trial of Ovarian Cancer Screening (UKCTOCS).

**Design:**

Prospective birth cohort analysis.

**Setting:**

Population cohort invited between 2001 and 2005 from age-sex registers of 27 Primary Care Trusts in England, Wales and Northern Ireland and recruited through 13 National Health Service Trusts.

**Participants:**

202 638 postmenopausal women aged 50–74 years at randomisation to UKCTOCS between April 2001 and October 2005.

**Interventions:**

Women were stratified into the following six birth cohorts (1925–1929, 1930–1934, 1935–1939, 1940–1944, 1945–1949, 1950–1955) based on year of birth. Self-reported data on reproductive factors provided at recruitment were explored using tabular and graphical summaries to examine for differences between the birth cohorts.

**Outcome measures:**

Trends in mean age at menarche and menopause, use of oral contraceptives, change in family size, infertility treatments, tubal ligation and hysterectomy rates.

**Results:**

Women born between 1935 and 1955 made up 86% of the cohort. Median age at menarche decreased from 13.4 for women born between 1925 and 1929 to 12.8 for women born between 1950 and 1955. Increased use of the oral contraceptives, infertility treatments and smaller family size was observed in the younger birth cohorts. Tubal ligation rates increased for those born between 1925 and 1945, but this increase did not persist in subsequent cohorts. Hysterectomy rates (17–20%) did not change over time.

**Conclusions:**

The trends seen in this large cohort are likely to reflect the reproductive history of the UK female postmenopausal population of similar age. Since these are risk factors for hormone-related cancers, these trends are important in understanding the changing incidence of these cancers.

**Trial registration number:**

International Standard Randomised Controlled Trial Number: 22488978.

**Strengths and limitations of this study**

- Largest birth cohort analysis to report on trends in reproductive and lifestyle factors.
- Over 25 000 women in each of the birth cohorts barring the earliest.
- Women born between the 1920s and 1950s were invited from population age-sex registers rather than self-referral.
- High-quality self-reported data on reproductive and lifestyle factors.
- Key limitations were that women were likely to be more health-conscious as they agreed to participate in a screening trial and a lack of details such as oral contraceptives formulation and type of infertility treatment.

## Introduction

In recent years, there has been increasing interest and mounting evidence on the long-term impact of reproductive and lifestyle factors on health, especially cancer, cardiovascular disease and overall mortality.^1^ The main focus has been oral contraceptives use, hormone replacement therapy (HRT), parity and breastfeeding.^2–4^ There are, however, little data examining how these factors have varied over time.

During the 20th century, data from the USA showed an increase in the reproductive lifespan of women from 36.1 years for those born between 1915 and 1919 to 37.7 years for those born between 1935 and 1939 as a result of earlier menarche and later menopause.^5^ A decline in age at menarche from 13.5 years in those born between 1908 and 1919 to 12.6 for those born between 1945 and 1949 has also been reported from the UK.^6^ Oral contraceptives use has increased in Europe^7^ and the USA^8^ and this has been accompanied by a decline in tubal ligation rates in the USA^9^ and Scandinavia.^10^ More recently, there have been reports from Italy, Australia and the UK on a reduction in hysterectomy rates over the past decade.^11–13^ All of these factors most likely affect a woman's lifetime exposure to circulating endogenous hormones and ultimately future disease/cancer burden.

In this paper, we report on trends in reproductive and lifestyle factors in British women born between 1925 and 1955 by undertaking a birth cohort analysis of participants in the UK Collaborative Trial of Ovarian Cancer Screening (UKCTOCS).

## Methods

### Study population and data collection

UKCTOCS is a randomised controlled trial designed to assess the impact of screening on ovarian cancer mortality. Between April 2001 and October 2005, 1.2 million women born between 1925 and 1955 were randomly invited from the age-sex registers of 27 participating primary care trusts. Women had to be postmenopausal (defined as either >12 months amenorrhoea following a natural menopause or hysterectomy, or >12 months of hormone replacement therapy started for menopausal symptoms) in addition to being aged 50–74 years at recruitment. Exclusion criteria included bilateral oophorectomy, previous ovarian malignancy, increased risk of familial ovarian cancer, active non-ovarian malignancy and participation in other ovarian cancer screening trials. A total of 288 955 agreed to participate in the trial and 202 638 eligible postmenopausal women were finally recruited through 13 regional trial centres located in National Health Service Trusts in England, Wales and Northern Ireland.^14^ The trial design, including details of recruitment and randomisation, has been described in detail elsewhere, which also included the detailed CONSORT diagram.^14^ All women provided written consent.

At recruitment, all women completed an 18-item questionnaire. The recruitment questionnaire (see online supplementary data, appendix 1) captured data on demographics (ethnicity), reproductive factors (age at menarche, previous oral contraceptives use and duration, age at menopause), gynaecological procedures (tubal ligation, hysterectomy with ovarian conservation) and parity (number of pregnancies <6 months which included miscarriages, abortions and ectopic pregnancies; number of pregnancies lasting over 6 months) and infertility treatment. Age at menopause was derived from age at last period excluding those who had self-reported hysterectomy at recruitment with a subgroup analysis excluding those who reported HRT use at recruitment. Personal history of cancer and family history of breast or ovarian cancer were also captured. For the purposes of this analysis, the women were grouped according to year of birth into the following six cohorts: 1925–1929, 1930–1934, 1935–1939, 1940–1944, 1945–1949 and 1950–1955. In a subset of 144 454 women who answered a postal follow-up questionnaire 3–5 years postrandomisation, information on education was also available.

10.1136/bmjopen-2016-011822.supp1 supplementary appendix

### Statistical analysis

Extreme biometric data values, which were almost certainly errors, were discarded to protect the analyses from their undue influence on statistical methods for continuous data. The data were logged and values that were more than 5 SDs from the mean were discarded. The mean and SD were then recalculated. This threshold rule was applied one more time using the recalculated summary statistics, before anti-logging the data back to the original metric. While this removed distorting values, it may also have excluded some genuine values that were simply extreme outliers as it was based on a statistical rule of deviation from the mean rather than a judgement on what constitutes an erroneous value.

Trends and differences in baseline characteristics such as age at menarche, age at menopause, oral contraceptives use, hysterectomy, tubal ligation, infertility treatment and parity were explored using tabular and graphical summaries with 95% CIs added to the latter. Formal tests for trends were not considered, as the trend was not linear for most variables. In addition, the large sample size resulted in almost all pairwise cohort-comparisons for each variable being highly significant even when adjusted for multiple comparisons.

## Results

The data captured at recruitment were available for 202 637 women as one woman asked for all her personal identifiers and associated data to be removed from the UKCTOCS database. All women were postmenopausal. The median age at completion of the baseline questionnaire (age at randomisation) was 60.6 (IQR 55.9–66.2).

### Demographics

Except for the oldest birth cohort (1925–1929), each of the remaining five cohorts included a minimum of 25 000 women (table 1). The majority (96.4%; 195 275) were white. When the women were separated into birth cohorts, there was an increase in the percentage of non-Caucasians in the younger compared to the oldest cohorts. However, it must be noted that the numbers in these groups were small (data not shown). About 40.0% of women in the oldest (1925–1929) cohort reported no formal educational qualification compared with 19.1% in the youngest birth cohort. This was paralleled by a rise in those with a college/university degree from 14.6% (1925–1929) to 29.5% (1950–1955) (data not shown). About 6.0% had a personal history of cancer with breast cancer being the most common (table 1).

**Table 1:** Baseline characteristics of the UKCTOCS cohort

	Number of women	Per cent
Birth cohort		
1925 to 1929	2588	1.3
1930 to 1934	26 201	12.9
1935 to 1939	41 418	20.4
1940 to 1944	51 057	25.2
1945 to 1949	55 510	27.4
1950 to 1955*	25 863	12.8
Ethnicity		
Caucasian	195 275	96.4
Black	2769	1.4
Asian	1856	0.9
Other	1695	0.8
Missing data	1043	0.5
Cancer history		
Personal history of cancer	12 060	6.0
Breast	7652	3.8
Bowel	760	0.4
Lung	100	0.1
Other	3548	1.8
Maternal history of cancer		
Maternal history of breast/ovarian cancer	16 025	7.9
Breast	12 990	6.4
Ovarian	3162	1.5
Both	127	0.1
Family history of breast or ovarian cancer		
Family history of ovarian cancer	9184	4.5
Number of relatives with ovarian cancer		
1	9028	0.1
2	142	0.1
>2	14	0
Family history of breast cancer	45 010	22.2
Number of relatives with breast cancer		
1	36 965	18.2
2	6460	3.2
>2	1585	0.8
BMI		
Underweight (<18.5)	1874	0.9
Normal (18.5–24.9)	83 062	41
Overweight (25.0–29.9)	74 283	36.7
Obese (>30)	41 236	20.3
Education†		
College/University or equivalent	29 428	20.2
Other formal education	69 862	48.4
No formal educational qualification	41 666	28.8
Missing data	3498	2.4

*Includes 370 women born in 1955

†Subcohort analysis of 144 454 from a follow-up questionnaire

BMI, body mass index; UKCTOCS, UK Collaborative Trial of Ovarian Cancer Screening.

### Menarche and menopause

**Figure 1:**
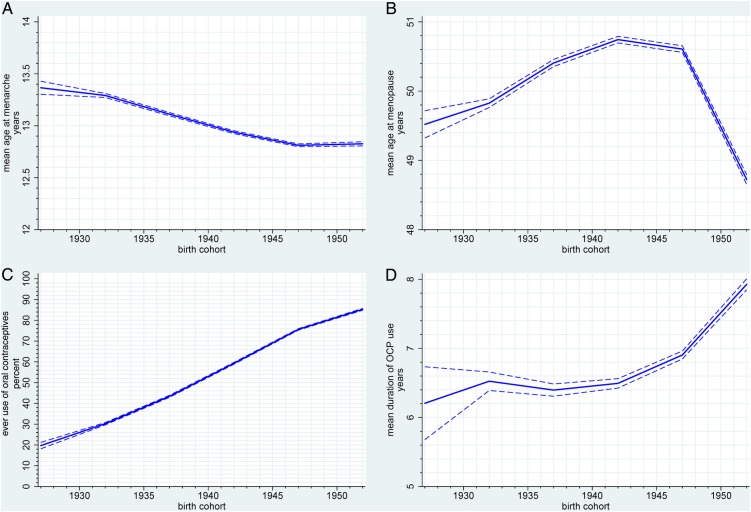
Trends in (A) age at menarche, (B) age at menopause, (C) ever use of oral contraceptives and (D) duration of oral contraceptives use in oral contraceptive users.

The mean age at menarche decreased significantly from 13.4 years for women born between 1925 and 1929 to 12.8 years for those born between 1950 and 1955 (figure 1A, see online supplementary data, appendix 2, supplementary table S1). The mean age at natural menopause (women who had hysterectomy at recruitment were excluded) for the cohort was 50.3 years (SD 4.32). On sensitivity analysis limited to those not using HRT at recruitment, the mean age at natural menopause was 50.1 years (SD 5.05). The mean age of natural menopause increased from 49.5 years for women born between 1925 and 1929 to 50.7 years for the 1940–1944 birth cohort (figure 1B, see online supplementary data, appendix 2, supplementary table S1) but then decreased to 48.7 years for the youngest birth cohort. Similar trends were observed when women on HRT were excluded.

10.1136/bmjopen-2016-011822.supp2 supplementary appendix

### Contraception and gynaecological procedures

**Figure 2:**
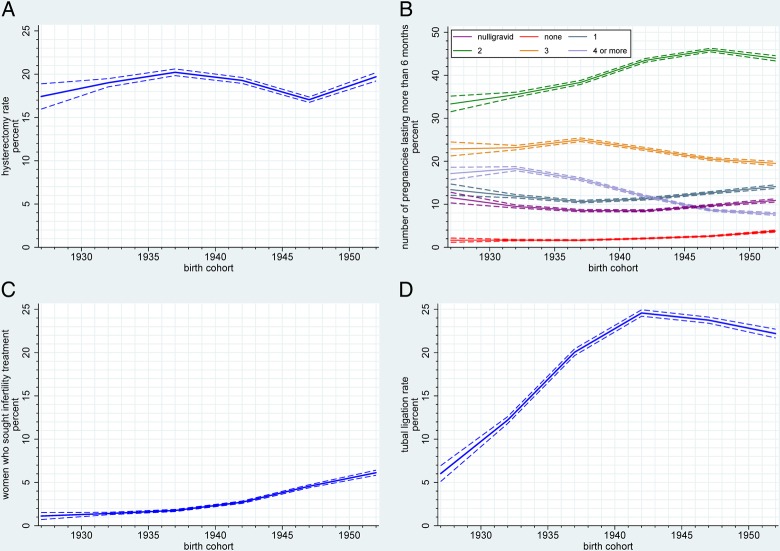
Trends in (A) hysterectomy rates between the birth cohorts (B) parity (pregnancies >6 months; includes those who had at least one pregnancy <6 months but had no pregnancy >6 months *red line* and those who were nulligravid *purple line*), (C) those seeking infertility treatment, (D) tubal ligation rates between the birth cohorts.

Increased oral contraceptives use was reported by younger birth cohorts with the highest use of 85.3% in women born between 1950 and 1955 (figure 1C, see online supplementary data, appendix 2, supplementary table S1). The mean duration of oral contraceptives use increased from 6.2 years in those born between 1925 and 1929 to 7.9 years in the younger cohorts (1950–1955) (figure 1D, see online supplementary data, appendix 2, supplementary table S1). Overall, 18.9% (38 211) of women underwent hysterectomy with ovarian conservation, with the highest rate of 20.2% in women born between 1935 and 1939 (figure 2A, see online supplementary data, appendix 2, supplementary table S1).

### Parity and infertility

The overall proportion of women having a viable (lasting over 6 months) pregnancy was 88.4% and remained unchanged between the birth cohorts (see online supplementary data, appendix 2, supplementary table S1). Family size based on number of viable pregnancies decreased across birth cohorts. Fewer women had four or more pregnancies lasting over 6 months in the younger (7.5%) when compared to the older (16.6%) cohorts while those having one or two pregnancies increased (47.0% and 58.2%, respectively) (figure 2B, see online supplementary data, appendix 2, supplementary table S1).

The proportion of women who had never conceived (nulligravid) ranged from 8.5% to 11.6% and showed an obvious quadratic trend (figure 2B, see online supplementary data, appendix 2, supplementary table S1). This was despite a 5.5-fold increase in self-reported infertility treatment, which increased from 1.1% (of women born between 1925 and 1929) to 6.1% (of those in the 1950–1955 cohort) (figure 2C, see online supplementary data, appendix 2, supplementary table S1). The percentage of nulligravid women who had availed of infertility treatment increased from 1.0% of those born between 1925 and 1930 to 11.5% of women born between 1945 and 1950 (data not shown). Tubal ligation rates peaked at 24.6% in those born between 1940 and 1944 compared with only 6.0% of the women born between 1925 and 1929 undergoing the procedure (figure 2D, see online supplementary data, appendix 2, supplementary table S1).

## Discussion

### Main findings

To the best of our knowledge, this is the largest birth cohort analysis to report on trends in reproductive and lifestyle factors in women born between the 1920s and 1950s. We found that in the UK, family size decreased in younger birth cohorts accompanied by an increase in oral contraceptives use. The initial increase in uptake of tubal ligation was not sustained. For women born between 1925 and 1955, increasing use of infertility treatments was not accompanied by a change in the proportion of nulligravid women. The hysterectomy rates were similar across the cohorts. Finally, we observed a decrease in the mean age at menarche. These changing trends directly contribute to a woman's exposure to endogenous and exogenous oestrogen and are likely to influence the incidence of hormonally dependent cancers.

### Strengths and limitations

A major strength is the overall size with over 25 000 women in each birth cohort except the earliest. Instead of self-referral, one in six women aged 50–74 years in the general population was randomly chosen from age-sex registers and sent a personal invitation to participate.^14^ While a healthy volunteer effect was noted in the cohort early on,^15^ this was not pronounced at the end of the trial with the mortality rate ratio between the control and screening arms being 0.99 for overall deaths and 1.00 for cancers other than ovarian/peritoneal.^16^ While the analysis is dependent on self-reporting which is affected by recall bias, especially in older women, the validity of self-reporting of these variables has been previously described in the literature^17–21^ including validation of self-reported hysterectomy in this cohort.^18^ Scanning and checking of data entry reduced transcription error while the limited extent of missing data ensured that such records could be discarded without concern of bias. Furthermore, a strength of our data is that the narrowness of the CIs resulting from such a large sample means that any notable change in trend could be interpreted as a real difference and not random perturbation of the data.

A limitation is that this cohort consists of women who wished to participate in a randomised controlled trial of ovarian cancer screening. As a result, they are likely to be more health-conscious.^22^ About 96.4% were white compared with 92.1% in the 2001 census.^23^ Additionally, we only captured limited data at recruitment and the need to keep the questionnaire brief meant that no details regarding oral contraceptives formulation, details of infertility treatment, age at childbirth or duration of breastfeeding were captured.

### Interpretation

The mean age at menarche decreased from 13.4 years for women born between 1925 and 1929 to 12.8 years for those born in the later cohorts (1950–1955), which is comparable to a recent British study, showing a decline in the mean age at menarche from 13.5 years for women born between 1908 and 1919 to 12.6 years for those born between 1945 and 1949.^6^ Other studies have similarly reported a decline.^5^ ^24^ The mean age of natural menopause was 50.1 years for non-HRT users, which is similar to that reported for Western countries.^25^ We did not observe an increase in the mean age at menopause as previously reported.^5^ ^26–28^ The two together are major determinants of endogenous hormone exposure, which is associated with osteoporosis, heart disease and cancer risk, as well as all-cause mortality.^1^ ^4^ ^29^ Early age at menarche has been linked to increased risk of breast, endometrial and ovarian cancer.^3^ ^30^ ^31^ A recent meta-analysis of over 100 000 women showed that the risk of breast cancer increased by lengthening of a woman's reproductive years and early menarche played a greater role than later menopause.^30^ While the women in the younger birth cohorts in our study had a lower age at menopause, this could be the result of the trial eligibility criteria that required women to be postmenopausal rather than a real effect of decrease in age at menopause in this group of women. It is possible that the length of the reproductive period may be a more important factor in predisposing a woman to risk of hormone-related cancers. Similar trends of earlier menarche and decrease in family size have also been reported for a large cohort of Chinese women.^32^

Oral contraceptives use was highest among women in the 1950–1955 cohort with 85% reporting ever use. Since the ‘pill’ was only discovered in the 1950s and approved for contraceptive use in the UK in 1961, women in the later birth cohorts may have had easier access to it. More importantly, changing social norms made its use more acceptable. By 1970, negative publicity surrounding the potential risks (mainly thrombosis) published in various British medical journals and newspapers may have influenced womens' decision against the use of oral contraceptives.^33^ However, we did not observe an impact within our cohort, as oral contraceptives use increased steadily. Overall, 60% had reported ever use, which is identical to the percentage of oral contraceptive users reported from the Million Women Study. A recent large US study on oral contraceptives and mortality found the prevalence of oral contraceptives use to be 48%, but the limitation of the US study is that it only included married women between the ages of 30–55 years.^2^

The increase in tubal ligation rates for women born until 1945 likely reflects the availability of the procedure and is most likely influenced by the change in ambulatory settings (inpatient to outpatient). However, the subsequent plateau may reflect increasing availability of other effective options for contraception. Overall, 19% underwent hysterectomy with conservation of at least one ovary. This is in keeping with a 20% hysterectomy rate at median age 60 years reported by a UK study which reported that most of these women had undergone the procedure for benign conditions in their mid-40s.^34^ The UKCTOCS rates do not include hysterectomies where both ovaries were removed as such women were ineligible to participate. This may explain the lower rates compared with 25.1% noted in the Million Women Study. About 7.4% of women in the latter cohort had undergone bilateral oophorectomy.^35^ There were small fluctuations in hysterectomy rates across the birth cohorts, which most likely relate to changing attitudes to ovarian conservation during hysterectomy and increasing trends in conservative management of dysfunctional uterine bleeding and fibroids.

There was a trend towards larger families in those born prior to 1940 in keeping with the postwar era in the UK (1945–1950) which saw sharp peaks in the total fertility rate during the 1960s Baby Boom.^36^ This was when women in the 1930–1940 cohorts were of childbearing age. Later cohorts tended to have two children, reflecting increased availability of family planning methods, especially oral contraceptives and changing social attitudes towards female education and employment. Women seeking infertility treatment increased steadily in later birth cohorts as previously reported.^37^ This possibly reflects the increasing availability of infertility treatments. Despite this, the proportion of women who never conceived remained static across the birth cohorts, as success rates were low prior to introduction of in vitro fertilisation and embryo transfer.^38^

The changes described in reproductive and lifestyle factors are likely to contribute to changing incidence in hormone-related cancers such as breast, endometrial and ovarian cancer. For ovarian cancer, for example, incidence rates in Great Britain have increased by 28% between 1979–1981 and 1997–1999 and then decreased by 8%.^39^ To forecast impact, statistical modelling needs to take into account both factors that decrease incidence such as increasing oral contraceptives use in younger birth cohorts as well as those that increase incidence such as smaller families, earlier menarche, late menopause and increasing life expectancy.

## Conclusion

There were clear differences in the reproductive factors across birth cohorts in the UK, with later birth cohorts reporting a lower age of menarche, smaller family size and increased use of oral contraceptives and infertility treatments and a decrease in the age at menopause in those born after 1945. While the absolute rates cannot be extrapolated to the general population, the trends in this large cohort of women, although influenced by a ‘healthy volunteer effect’, most likely represent trends in the UK population. The changes in lifetime sex hormone exposure resulting from these changes could in part explain the trends in breast, endometrial and ovarian cancer incidence as well as other diseases such as osteoporosis, heart disease and neurodegenerative disorders. Further exploration of these trends could explain their more precise contribution to both disease incidence and mortality.
