# Supplementary material for: Changing trends in reproductive/lifestyle factors in UK women: descriptive study within the UK Collaborative Trial of Ovarian Cancer Screening (UKCTOCS)
Source: BMJ Open. 2017 Mar 6;7(3):e011822. doi: 10.1136/bmjopen-2016-011822 (PMC5353253; doi:10.1136/bmjopen-2016-011822)
Supplement: supplementary appendix [file bmjopen-2016-011822supp_appendix1.pdf]

United Kingdom Collaborative Trial of Ovarian Cancer Screening (UKCTOCS)

Please complete this form in BLACK INK and in BLOCK CAPITALS. It will help us check that you are eligible for the study as well as collect some data regarding your risk of developing ovarian cancer. If the personal or GP details are incorrect please insert the correct details into the "Amended details" box.

Your Personal Details

Amended details

Your GP Details

Dr

NHS No. **D.o.B.** **AP** **ID**

Your Home Telephone No.

Your Work Telephone No.

ELIGIBILITY DETAILS (use black ink and BLOCK CAPITALS or place a cross "X" in the appropriate boxes)

1. When was your last period? **(dd/mm/yyyy)** / /

2. Are you currently on Hormone Replacement Therapy (HRT)? Yes ☐ No ☐

   If Yes then when did you start taking HRT? **(dd/mm/yyyy)** / /

3. Have you had both your ovaries removed? Yes ☐ No ☐

4. Have you ever had cancer diagnosed (except skin cancer)? Yes ☐ No ☐ Go to Q5

   If yes, what cancer was it?

   ☐ Ovary ☐ Breast ☐ Bowel ☐ Lung ☐ Other

   When was it diagnosed? **(dd/mm/yyyy)** / /

5. Have you had any treatment for any cancer (including surgery, chemotherapy, radiotherapy) in the last 12 months(not including tamoxifen)? Yes ☐ No ☐

4999524552

6. If any of the following relatives have had **OVARIAN CANCER** please write the number of affected relatives in the approprate box. Please enter 0 for no affected relatives. **(e.g. 0 Mother, 2 Sister, 0 Daughter).**

☐ Mother ☐ Daughter ☐ Sister ☐ Aunt ☐ GrandMother ☐ GrandDaughter

7. If any of the following relatives have had **BREAST CANCER** please write the number of affected relatives in the approprate box. Please enter 0 for no affected relatives. **(e.g. 0 Mother, 2 Sister, 1 Daughter).**

☐ Mother ☐ Daughter ☐ Sister ☐ Aunt ☐ GrandMother ☐ GrandDaughter

8. Are you currently taking part in any other ovarian cancer screening trial? Yes ☐ No ☐

If yes what is your study reference number? ☐☐☐☐☐☐☐☐☐☐

ADDITIONAL INFORMATION

ID ☐

9. Your height (cm) ☐☐☐ Your weight (kg) ☐☐☐

Or (in) ☐☐☐ Or (lb) ☐☐☐

10. Country of birth (please place an "X" as appropriate)

England ☐ Northern Ireland ☐ Scotland ☐ Irish Republic ☐ Wales ☐ Elsewhere ☐

11. Ethnic group, please place an "X" in the appropriate box. (If you are descended from more than one ethnic or racial group, please sellect the group you consider you belong to or choose "Any other ethnic origin")

☐ White ☐ Indian ☐ Pakistani ☐ Chinese ☐ Bangladeshi

☐ Black-African ☐ Black-Caribbean ☐ Black-other ☐ Any other ethnic origin

12. At what age did you first have your period? ☐☐

13. How many pregnancies have you had which ended before they reached 6 months (including miscarriages, ectopic pregnancies)? ☐☐

14. How many pregnancies have you had which lasted beyond 6 months (including all deliveries - both term and preterm)? ☐☐

15. Have you ever taken the oral contraceptive pill? Yes ☐ No ☐

If yes, how many years in total did you take the pill? Years ☐☐

16. Have you ever had a hysterectomy (removal of the womb)? Yes ☐ No ☐

17. Have you had a sterilisation operation (To block your tubes)? Yes ☐ No ☐

18. Have you ever had any treatment for infertility? Yes ☐ No ☐
