# Supplementary material for: Changing trends in reproductive/lifestyle factors in UK women: descriptive study within the UK Collaborative Trial of Ovarian Cancer Screening (UKCTOCS)
Source: BMJ Open. 2017 Mar 6;7(3):e011822. doi: 10.1136/bmjopen-2016-011822 (PMC5353253; doi:10.1136/bmjopen-2016-011822)
Supplement: supplementary appendix [file bmjopen-2016-011822supp_appendix2.pdf]

Supplementary Table 1

Reproductive factors	Birth Cohort											
	1925 to 1929		1930 to 1934		1935 to 1939		1940 to 1944		1945 to 1950		1950 to 1955 [a]	
	No. 2,588	%	No. 26,201	%	No. 41,418	%	No. 51,057	%	No. 55,510	%	No. 25,863	%
Ever use of Oral contraceptive pill (OCP)	510	19.7%	7920	30.2%	17988	43.4%	30280	59.3%	41978	75.6%	22061	85.3%
Tubal ligation	156	6.0%	3199	12.2%	8305	20.1%	12549	24.6%	13184	23.8%	5742	22.2%
Hysterectomy	451	17.4%	4981	19.0%	8374	20.2%	9844	19.3%	9466	17.1%	5095	19.7%
Infertility	29	1.1%	370	1.4%	729	1.8%	1387	2.7%	2530	4.6%	1584	6.1%
Never pregnant	299	11.6%	2488	9.5%	3532	8.5%	4335	8.5%	5387	9.7%	2819	10.9%
Ever pregnant[b]	2275	88.4%	23624	90.5%	37783	91.5%	46595	91.5%	50017	90.3%	22982	89.1%
No pregnancies ≥6 months	46	1.8%	448	1.7%	705	1.7%	1080	2.1%	1468	2.6%	985	3.8%
Pregnancies ≥6 months[c]	2229	86.6%	23176	88.8%	37078	89.7%	45515	89.4%	48549	87.6%	21997	85.3%
1	*347*	*13.5%*	*3112*	*11.9%*	*4399*	*10.6%*	*5758*	*11.3%*	*7041*	*12.7%*	*3651*	*14.2%*
2	*863*	*33.5%*	*9301*	*35.6%*	*15885*	*38.4%*	*22203*	*43.6%*	*25460*	*46.0%*	*11367*	*44.1%*
3	*592*	*23.0%*	*6072*	*23.3%*	*10357*	*25.1%*	*11661*	*22.9%*	*11394*	*20.6%*	*5046*	*19.6%*
≥4	*427*	*16.6%*	*4691*	*18.0%*	*6437*	*15.6%*	*5893*	*11.6%*	*4654*	*8.4%*	*1933*	*7.5%*
	**Mean**	**SD**	**Mean**	**SD**	**Mean**	**SD**	**Mean**	**SD**	**Mean**	**SD**	**Mean**	**SD**
Age at menarche	13.4	1.6	13.3	1.7	13.1	1.6	13.0	1.6	12.8	1.6	12.8	1.6
Age at menopause	49.5	5.3	49.8	5.2	50.4	5.1	50.7	4.7	50.6	3.8	48.7	3.3
Duration of OCP use (years) in those reporting ever use of OCP	6.2	5.8	6.5	5.9	6.4	5.8	6.5	5.8	6.9	6.0	7.9	6.4

Footnote: [a] Includes 370 women born in 1955; [b] any pregnancy <6 or ≥6 months; [c] includes those with pregnancy <6 months

percentages based on those with complete data on pregnancy
